# Probiotic Potential, Genomic Characterization, and In Silico Insights of Five *Lactiplantibacillus plantarum* Strains Isolated from Fermented Cacao Beans Against Multidrug-Resistant *Pseudomonas aeruginosa*

**DOI:** 10.3390/antibiotics15040334

**Published:** 2026-03-26

**Authors:** Phoomjai Sornsenee, Nawanwat C. Pattaranggoon, Pinkanok Suksabay, Yosita Leepromma, Conny Turni, Chonticha Romyasamit

**Affiliations:** 1 Department of Family and Preventive Medicine, Faculty of Medicine, Prince of Songkla University, Songkhla 90110, Thailand; ezipnary@gmail.com; 2 CPF Food Research and Development Center, Wang Noi District, Phra Nakhon Si Ayutthaya 13170, Thailand; nawanwat.pa@gmail.com; 3 College of Graduate Studies, Walailak University, Nakhon Si Thammarat 80160, Thailand; pinkanok.su@mail.wu.ac.th (P.S.); yosita.le@mail.wu.ac.th (Y.L.); 4 Centre for Animal Science, Queensland Alliance of Agriculture and Food Innovation, The University of Queensland, Brisbane, QLD 4072, Australia; c.turni1@uq.edu.au; 5 Department of Medical Technology, School of Allied Health Sciences, Walailak University, Nakhon Si Thammarat 80160, Thailand; 6 Research Center in Tropical Pathobiology, Walailak University, Thasala District, Nakhon Si Thammarat 80160, Thailand; 7 Center of Excellence in Innovation of Essential Oil and Bioactive Compounds, Walailak University, Nakhon Si Thammarat 80160, Thailand

**Keywords:** lactic acid bacteria, postbiotics, *Pseudomonas aeruginosa*, whole-genome analysis, antimicrobial activity

## Abstract

**Background/Objectives:** Severe and recurrent infections due to multidrug-resistant (MDR) *Pseudomonas aeruginosa* necessitate alternative antimicrobial strategies. Fermented cacao beans represent a niche microbial ecosystem with the potential to harbor beneficial lactic acid bacteria (LAB). This study aimed to isolate and characterize LAB strains from fermented cacao beans in southern Thailand and to evaluate their probiotic potential and antimicrobial activity against MDR *P. aeruginosa*. **Methods and Results:** Five *Lactiplantibacillus plantarum* isolates were identified via MALDI-TOF MS and whole-genome sequencing (WGS). All strains demonstrated antimicrobial activity against 17 clinical MDR *P. aeruginosa* isolates and CR14 exhibited the largest inhibition zone. The isolates displayed robust probiotic traits, including survival under simulated gastrointestinal conditions. Acid tolerance (pH 2.0) reached 61.15 ± 7.75%, while resistance to pepsin, pancreatin, and bile salts exceeded 88%, 91%, and 92%, respectively. Strong adhesion was confirmed via auto-aggregation (55.02 ± 1.75%), hydrophobicity (45.58 ± 0.96%) and Caco-2 cell attachment (up to 98.11 ± 3.28%). WGS revealed multiple plantaricin-encoding clusters. Coarse-grained molecular dynamic simulations showed that two-peptide plantaricins (*plnJ/K* and *plnNC8-αβ*) self-assembled and formed stable pores in bacterial membrane models, confirming a pore-forming antimicrobial mechanism. The strains lacked acquired resistance genes and virulence factors, confirmed by in silico safety assessments. **Conclusions:** Thus, these *L. plantarum* strains are promising probiotics for managing MDR *P. aeruginosa* via functional foods or adjunct therapies.

## 1. Introduction

Antimicrobial resistance (AMR) has become one of the most critical global health challenges of the 21st century, underscoring the urgent need for novel therapeutic strategies. In 2024, the World Health Organization (WHO) updated its Bacterial Priority Pathogens List (BPPL), in which *Pseudomonas aeruginosa* (specifically the carbapenem-resistant strains) was categorized as a critical-priority pathogen [1]. *P. aeruginosa* is a rod-shaped, non-spore-forming, Gram-negative bacterium that remains a significant global concern due to its high incidence in both community-acquired and hospital-acquired infections. It is particularly prevalent in bloodstream infections, respiratory tract infections, cystic fibrosis-associated complications, soft-tissue wound infections, urinary tract infections (UTI), and eye infections, and continues to be among the most burdensome bacterial threats worldwide [2,3]. According to a 2019 global investigation, *P. aeruginosa* caused about 559,000 deaths globally (95% UI: 389,000–819,000), among which 233,000 were associated with lower respiratory tract infections and 163,000 with bloodstream infections [4]. Furthermore, over 18.3 million disability-adjusted life years were attributed to illnesses caused by this pathogen in 2019 [4]. The growing prevalence of antimicrobial resistance, especially the emergence of multidrug-resistant (MDR) *P. aeruginosa*, which severely restricts available therapeutic options and is strongly linked to increased patient mortality, exacerbates this already significant burden [5].

The major factor contributing to the pathogenicity of *P. aeruginosa* is its ability to form biofilms, which are structured microbial communities embedded within an extracellular polymeric substance (EPS) matrix that protects cells from antibiotics and host immune defenses [6]. Antibiotic tolerance in biofilm-associated cells can be significantly higher than in planktonic cells. Moreover, *P. aeruginosa* regulates virulence and biofilm development through coordinated quorum-sensing systems, including the *lasI/lasR*, *rhlI/rhlR*, and *pqsA–E* regulatory circuits. The stability and persistence of biofilms are further supported by key biofilm-associated genes, such as *pel*, *psl*, and *algD* [7]. These mechanisms make *P. aeruginosa* infections highly persistent, chronic, and difficult to eradicate.

Carbapenems, fluoroquinolones, and aminoglycosides remain the standard therapeutic options for treating *Pseudomonas aeruginosa* infections; however, their effectiveness is increasingly compromised by high levels of antimicrobial resistance, leading to frequent treatment failures and recurrent infections [3]. Consequently, there is growing interest in alternative therapeutic and preventive strategies, including herbal medicines, bacteriophage therapy, antimicrobial peptides, extracellular vesicles, and probiotics [8,9].

Probiotics are defined as “live microorganisms that, when administered in adequate amounts, confer a health benefit on the host” [10]. Sources of probiotics are generally classified into two main groups: dairy-based products and non-dairy products such as fermented foods, including fermented cacao beans [11]. The fermentation of cacao beans, a critical step in chocolate production, involves a complex succession of microorganisms, including yeasts, acetic acid bacteria, and lactic acid bacteria (LAB) [9]. The acidic and high-sugar environment of the cacao pulp creates a selective niche that supports the growth of LAB, many of which are recognized for their probiotic potential [12,13]. LAB are a group of Gram-positive microorganisms, widely regarded as safe (generally recognized as safe; GRAS), and are characterized by the production of lactic acid as their primary fermentation end product. They have a long history of safe use in food fermentation. Among them, *Lactiplantibacillus plantarum* (formerly *Lactobacillus plantarum*) is of particular interest because of its exceptional ecological versatility and ability to thrive in a wide range of environments, including the human gastrointestinal tract [14].

Bacteriocins are ribosomally synthesized antimicrobial peptides produced by bacteria that inhibit the growth of closely related or pathogenic microorganisms. These peptides are typically classified into several groups based on their structure, molecular weight, and post-translational modifications, including class I antibiotics, class II small heat-stable peptides, and class III larger heat-labile proteins [15]. *L*. *plantarum* is known to produce a diverse range of bacteriocins collectively referred to as plantaricins and are recognized for their antimicrobial activity against foodborne and clinical pathogens. These bacteriocins often function through membrane disruption or pore formation, contributing to the antagonistic activity of *L. plantarum* against competing microorganisms [16].

Several studies have demonstrated that *L. plantarum* strains possess antagonistic activity against a wide range of pathogenic microorganisms [17,18]. These antagonistic effects are typically multifactorial and include competitive exclusion for nutrients and adhesion sites, modulation of host immune responses, and the production of antimicrobial compounds such as organic acids, hydrogen peroxide, and bacteriocins [14]. Moreover, *L. plantarum* KCCP11226, which produces 4,4′-diaponeurosporene as a natural antioxidant, has been proposed as a potential functional probiotic [19]. However, the probiotic potential of *L. plantarum* strains isolated from fermented cacao beans in southern Thailand, particularly their ability to inhibit MDR *P. aeruginosa* and disrupt biofilm formation, remains underexplored. Therefore, this study aimed to investigate the probiotic properties and genomic characteristics of five *L. plantarum* strains isolated from fermented cacao beans, with a specific focus on their antimicrobial and antibiofilm activities against MDR *P. aeruginosa.*

## 2. Results

### 2.1. Bacterial Isolation and Identification

Five isolates of *L. plantarum* (CR02, CR09, CR10, CR13, and CR14) were obtained from fermented cacao beans collected at local farms in Nakhon Si Thammarat Province, southern Thailand. All isolates showed the typical characteristics of LAB, appearing as Gram-positive rods and testing negative for catalase. Species identification using matrix-assisted laser desorption/ionization time-of-flight mass spectrometry (MALDI-TOF MS) showed scores of >2.00, indicating a high-confidence identification (Supplementary Table S2). Final confirmation was obtained by short-read whole-genome sequencing (WGS) on the MGISEQ-2000 platform, confirming that all isolates belonged to *L. plantarum*.

### 2.2. Antimicrobial Activity

All five *L. plantarum* isolates showed inhibitory effects against *P. aeruginosa* ATCC 15692 and all 17 clinical MDR *P. aeruginosa* isolates (Using the agar well diffusion assay). The inhibition zones varied among the strains, with CR09, CR10, and CR14 producing larger zones against several isolates. CR14 showed the highest inhibition against *P. aeruginosa* ATCC 15692 (21.00 ± 0.00 mm), followed by CR09 (20.67 ± 0.58 mm) and CR10 (20.00 ± 1.73 mm). Similar patterns were observed across most clinical isolates (Table 1). These results suggest that *L. plantarum* isolated from fermented cacao beans exhibits broad-spectrum antimicrobial activity against MDR *P. aeruginosa*.

### 2.3. Characterization of Probiotic Properties

#### 2.3.1. Simulation of Gastrointestinal Tract (GIT) Tolerance

The survival of the five *L. plantarum* isolates was evaluated under simulated GIT conditions, including exposure to low pH (pH 2, 3, 4, and 7), pepsin, pancreatin, and bile salts (Table 2). All isolates survived acidic environments, although viability decreased markedly at pH 2 and 3. The survival rates at pH 2 ranged from 12.78 ± 4.10% to 61.15 ± 7.75%, with CR14 showing the highest acid tolerance (61.15 ± 7.75%), followed by CR02 (14.38 ±5.00) and CR10 (17.22 ± 2.55). At pH 3, survival ranged from 15.89 ± 7.03% (CR13) to 29.57 ± 5.07% (CR02)). When exposed to pH 4, the isolates showed greater tolerance, with survival rates ranging from 21.12 ± 1.11% (CR09) to 80.65 ± 5.38% (CR02). The highest survival was observed at pH 7, where all strains maintained >83% viability, with CR09 showing the highest survival (98.61 ± 2.40%).

In the presence of pepsin for 3 h, all isolates showed high viability, with survival rates ranging from 88.08% to 96.12%, indicating strong tolerance to simulated gastric digestion. Similarly, all isolates demonstrated strong survival when exposed to pancreatin for 4 h, with survival rates ranging from 91.37% to 99.88%, with CR14 and CR13 showing the highest tolerance.

Exposure to 0.3% bile salts for 4 h demonstrated that all isolates had high bile tolerance, with survival rates ranging from 92.79 ± 1.03 to 98.98 ± 0.80. CR14 exhibited the highest bile resistance, followed by CR13 and CR10, suggesting that these strains possess robust physiological mechanisms for coping with bile stress. These results confirm the ability of the five *L. plantarum* isolates to tolerate GIT conditions, highlighting *L. plantarum*’s probiotic potential.

#### 2.3.2. Auto-Aggregation and Enhancement of Adhesion Ability

**Figure 1:**
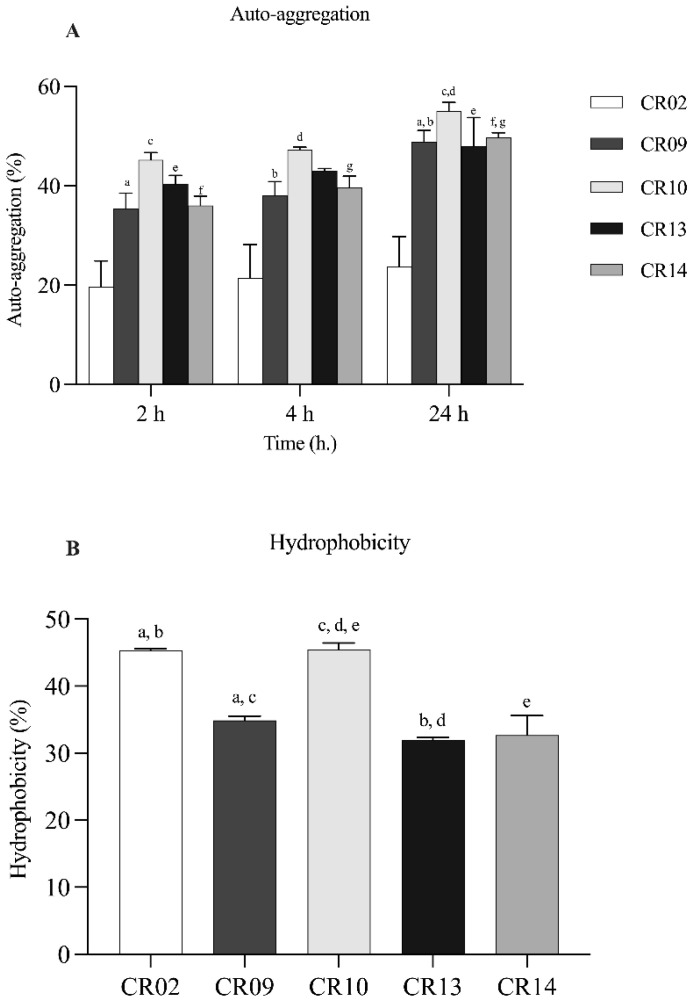
Surface properties of *Lactiplantibacillus plantarum* strains. (**A**) Auto-aggregation percentage measured at 2, 4, and 24 h. (**B**) Cell surface hydrophobicity percentage. Data represent mean ± standard deviation (SD) of three independent experiments. Different lowercase letters above bars indicate statistically significant differences between strains (*p* < 0.05).

**Figure 2:**
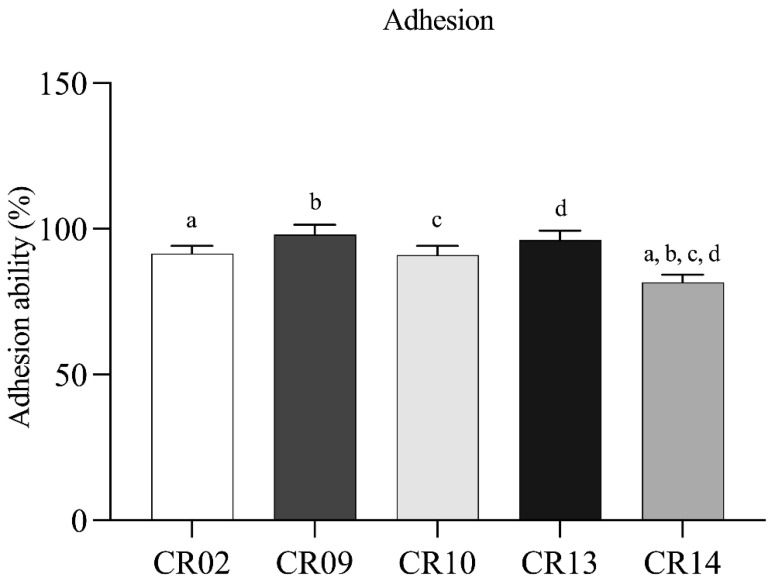
Adhesion ability of *L. plantarum* strains to Caco-2 intestinal epithelial cells. Results are expressed as the percentage of adherent bacteria relative to the initial inoculum. Data represent mean ± SD from three independent experiments. Different lowercase letters indicate statistically significant differences among strains (*p* < 0.05).

**Figure 3:**
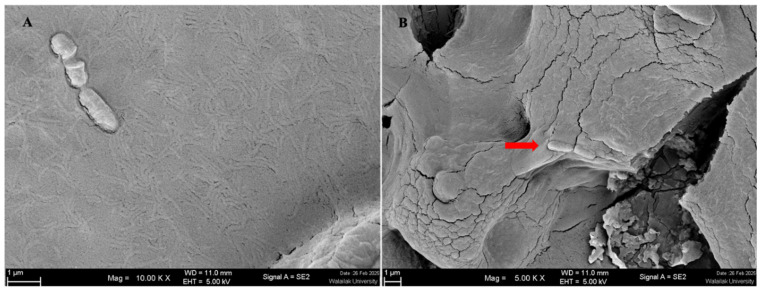
Scanning electron microscopy (SEM) analysis of bacterial interactions. (**A**) Morphology of *L. plantarum* cells in pure culture. (**B**) *L. plantarum* adhering to the microvilli of the Caco-2 cell monolayer. The red arrow indicates the attachment of bacterial cells to the epithelial surface. Magnification: 10,000× (**A**) and 5000× (**B**).

The auto-aggregation abilities of the five *L. plantarum* isolates were assessed at 2, 4, and 24 h at 37 °C. Auto-aggregation increased over time for all isolates. At 2 h, the isolates showed auto-aggregation values ranging from 19.69 ± 5.19% to 45.23 ± 1.44%, which increased to 21.49 ± 6.67% to 47.24 ± 0.54% at 4 h. After 24 h, the values further increased, ranging from 23.75 ± 5.60% to 55.02 ± 1.75%. Among all strains, *L. plantarum* CR10 exhibited the highest auto-aggregation after 24 h (55.02 ± 1.75%), followed by CR14 (49.72 ± 0.95%) and CR13 (48.00 ± 5.74%) (Figure 1A). The isolates also showed varying levels of cell surface hydrophobicity, with percentages ranging from 31.97 ± 0.38% to 45.58 ± 0.96% (Figure 1B). CR10 demonstrated the highest hydrophobicity (45.58 ± 0.96%), followed by CR02 (45.30 ± 0.25%) and CR09 (34.89 ± 0.62%). Adhesion to Caco-2 intestinal epithelial cells revealed that all five isolates exhibited strong attachment, with adhesion percentages ranging from 81.65 ± 2.72% to 98.11 ± 3.28%. CR09 showed the highest adhesion ability. Its adhesion rate was not significantly different from that of most strains, except for CR14 (Figure 2). Scanning electron microscopy (SEM) further confirmed the close physical interaction between the *L. plantarum* cells and the Caco-2 monolayer (Figure 3).

#### 2.3.3. Antibiotic Susceptibility and Hemolysis

None of the *L. plantarum* isolates showed β-hemolysis on BA, indicating that all strains were non-hemolytic and safe for potential probiotic use. Antibiotic susceptibility testing revealed a similar resistance pattern among the five isolates (Supplementary Table S3). All strains were resistant to ampicillin, vancomycin, clindamycin, kanamycin, erythromycin, and streptomycin, while being susceptible to gentamicin, tetracycline, and chloramphenicol.

#### 2.3.4. Characterization of Antimicrobial Substances

The antimicrobial activity of CFS from five isolates were evaluated using an agar well diffusion assay against pathogenic indicator strains. All isolates exhibited inhibitory activity under untreated conditions. Following pH neutralization (pH 7.0), the antimicrobial activity was reduced or completely abolished, indicating that organic acids play a major role in the observed inhibition. In contrast, treatment with proteinase K (1 mg/mL) resulted in a slight reduction in inhibitory activity in isolates 0601 and 0602, suggesting a possible minor contribution of proteinaceous compounds. However, the overall antimicrobial effect appears to be predominantly acid-mediated.

### 2.4. Genome Characteristics, Functional Analysis, and Antimicrobial Substances

**Figure 4:**
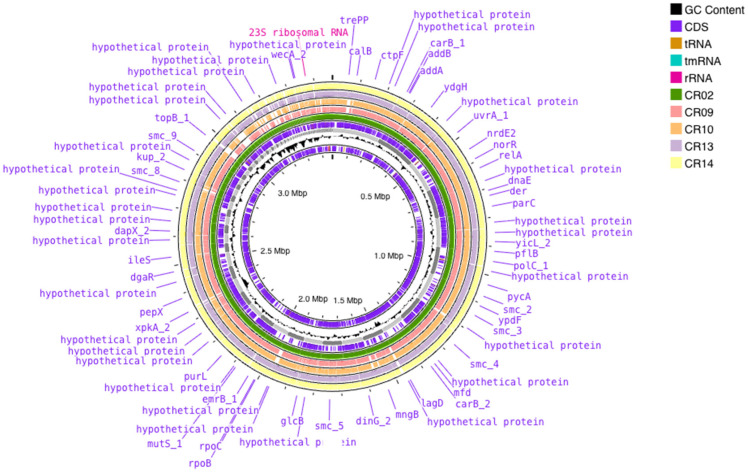
Circular genome maps of the five *L. plantarum* strains.

The genomic features of the five *L. plantarum* strains are summarized in Table 3. Genome sizes ranged from 3,232,214 bp to 3,465,846 bp, with CR02 being the largest genome and CR09 being the smallest. The GC content was highly conserved among the isolates, varying only slightly between 44.14% and 44.48%. Assembly statistics indicated differences in genome continuity across the strains. N50 values ranged from 101,367 bp for CR02 to 483,721 bp for CR14. The total number of contigs varied considerably, from 21 contigs in CR09 to 154 contigs in CR02. Genome annotation revealed that the number of coding sequences (CDS) ranged from 3036 in CR10 to 3311 in CR02. All genomes contained a single copy of the *tmRNA* gene. The number of rRNA and tRNA genes varied slightly among isolates, with 2–4 copies of rRNA genes and 50–61 tRNA genes detected (Table 3 and Figure 4). Prophage prediction identified multiple prophage regions in all strains. CR02 carried the highest number with eight prophage regions, whereas CR09, CR10, CR13, and CR14 each harbored five prophage regions (Supplementary Table S4). No plasmids were detected in any of the genomes.

In addition to general genomic features, specific survival and functional genes were analyzed (Table 4). The strains exhibited a highly conserved core of stress-response mechanisms. All five isolates possessed the complete F1F0-ATPase operon (*atpA-H*) for acid tolerance, key bile salt tolerance genes (*murE*, *mleS*), and major heat shock proteins (*hrcA*, *dnaK*, *clpB*). However, distinct variations were observed in cell wall and biofilm-associated genes. CR02 was the only strain to possess the *pbpX* gene, which was associated with gastrointestinal survival but was unique in lacking the *luxS* gene, a key regulator of quorum sensing and biofilm formation, which was present in all other strains. Furthermore, the *tagE* gene, involved in cell wall teichoic acid synthesis, was identified in CR02, CR09, and CR10 but was absent in CR13 and CR14.

Genome mining using the BAGEL4 platform identified several bacteriocin-encoding gene clusters across the strains (Supplementary Figure S1). Although all isolates encoded plantaricin-like peptides, the bacteriocin profiles varied among strains: CR02 encoded six bacteriocin genes: *plnA*, *plnK*, *plnJ*, *plnN*, *plnE*, and *plnF*. CR09 encoded four genes: *plnA*, *plnK*, *plnJ*, and *plnN*. CR10 encoded *plnJ*, *plnK*, *plnNC8-β*, and *plnNC8-α*. CR13 encoded six genes: *plnK*, *plnJ*, *plnN*, *plnF*, *plnA*, and *plnE*. CR14 carried six genes: *plnN*, *plnJ*, *plnK*, *plnE*, *plnF*, and *plnA*.

These findings indicate that cacao-derived *L. plantarum* strains harbor diverse bacteriocin-related gene clusters, including class II bacteriocins such as plantaricin A, EF, JK, N, and NC8 variants. However, most plantaricins are primarily active against Gram-positive bacteria, and their direct activity against Gram-negative pathogens remains limited due to the protective outer membrane barrier. Therefore, the antimicrobial activity observed against MDR *P. aeruginosa* in this study may also involve other antimicrobial metabolites produced by *L. plantarum*, such as organic acids or hydrogen peroxide, rather than bacteriocins alone.

### 2.5. In Silico Safety Evaluation

A comprehensive genomic safety assessment was conducted for the five *L. plantarum* isolates. Screening with ResFinder revealed no acquired antimicrobial resistance genes (ARGs) in any of the genomes, supporting the phenotypic susceptibility profiles, in which all strains were susceptible to erythromycin, tetracycline, and chloramphenicol but intrinsically resistant to vancomycin and aminoglycosides (Supplementary Table S2) Evaluation using the Virulence Factor Database (VFDB) showed no virulence-associated genes, indicating that the genomes lacked key determinants typically involved in pathogenicity, host damage, or evasion of host immunity. Moreover, the isolates were analyzed using PathogenFinder 2.0, which predicted no pathogenic potential for all five strains, consistent with the GRAS/QPS status of *L. plantarum* and its long history of safe use in fermented foods. Mobile genetic element analysis showed that all genomes contained prophage regions; however, these regions were predominantly incomplete or questionable, except for a few intact phages (Supplementary Table S3). Importantly, the prophages did not encode virulence factors, ARGs, or other elements associated with horizontal transfer of harmful traits. No plasmids were detected. Insertion sequence (IS) elements identified across the genomes were limited and did not contain accessory genes related to resistance, virulence, or mobilization, suggesting a low risk of gene transfer.

### 2.6. Molecular Dynamics Simulations

**Figure 5:**
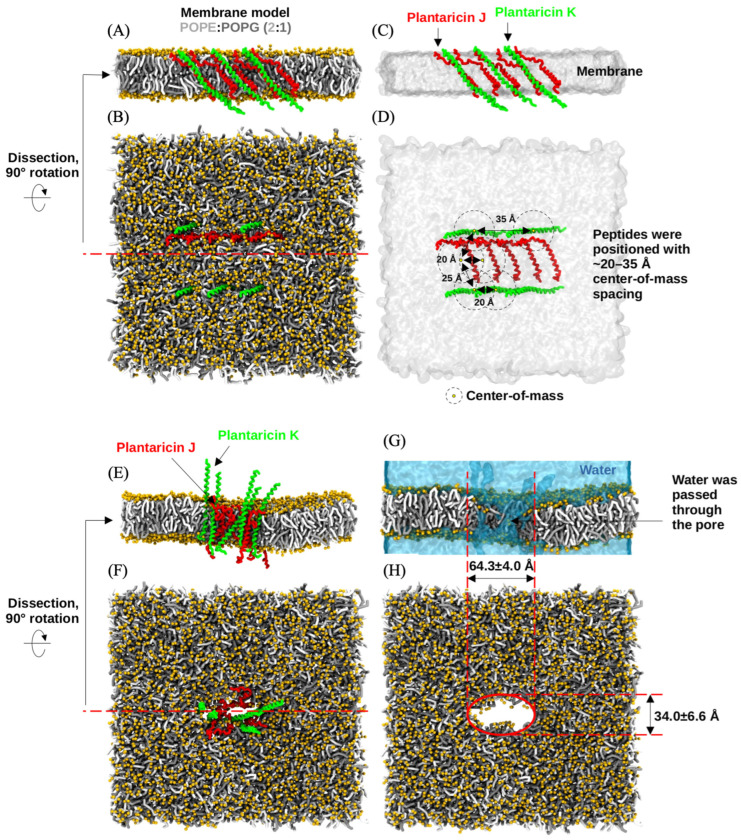
Coarse-grained molecular dynamics simulation of the Pln J/K system. (**A**–**D**) Initial system setup at 0 μs, depicting the initial spacing and orientation of Plantaricin J and K peptides within the POPE:POPG bilayer. (**E**–**H**) Final system state at 4 μs, showing the formation of a large pore structure (64.3 ± 4.0 Å × 34.0 ± 6.6 Å) and the resulting permeation of water molecules.

**Figure 6:**
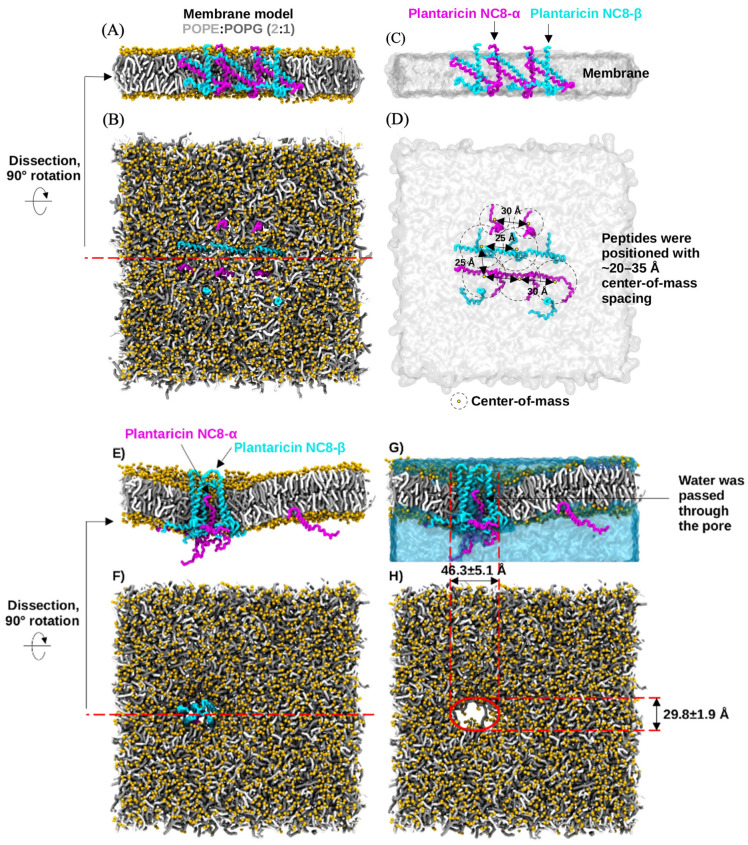
Coarse-grained molecular dynamics simulation of the Pln NC8-α/β system. (**A**–**D**) Representations of the initial peptide distribution at 0 μs. (**E**–**H**) State of the system after 4 μs, highlighting the assembly of the NC8-α/β complex and the measurement of a 46.3 ± 5.1 Å × 29.8 ± 1.9 Å pore.

**Figure 7:**
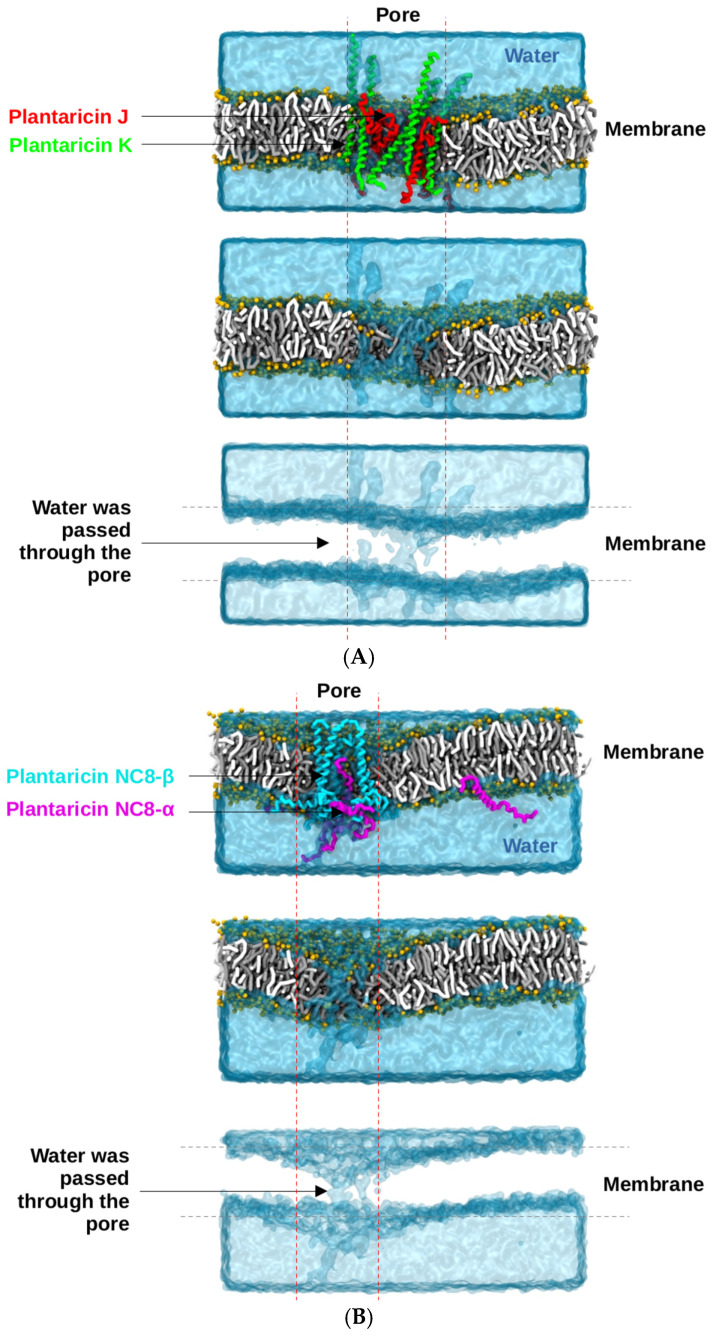
Comparative cross-sectional analysis of pore-mediated water transport. (**A**) Visualization of the water channel established by the Pln J/K complex. (**B**) Cross-sectional view of the Pln NC8-α/β system, illustrating the continuous water passage through the peptide-lined pore.

The coarse-grained (CG) molecular dynamics simulations, conducted over a cumulative sampling period of 4 μs, revealed the dynamic behavior of two-peptide bacteriocin systems within a bacterial membrane model. Both Plantaricin J/K (Pln J/K) and Plantaricin NC8-α/β (Pln NC8-α/β) successfully transitioned from their initial distributed states into organized pore-forming complexes. The Pln J/K system was characterized by the aggregation of the ten embedded peptides within the POPE:POPG (2:1) bilayer. Initially positioned as having center-of-mass distances of 20–35 Å (Figure 5), the peptides reorganized to form a stable transmembrane pore by the end of the simulation. The resulting pore exhibited an elliptical geometry with a primary axis of 64.3 ± 4.0 Å and a secondary axis of 34.0 ± 6.6 Å, as shown in Figure 5G,H. The Pln NC8-α/β system also demonstrated effective membrane disruption (Figure 6). This structural disruption facilitated the continuous passage of water molecules through the pore of the membrane (Figure 7A). The pore formed by Pln NC8-α/β was more compact than the Pln J/K system, measuring 46.3 ± 5.1 Å by 29.8 ± 1.9 Å (Figure 6G,H). Despite the smaller dimensions, the pore remained functional, maintaining a channel for water translocation across the bilayer (Figure 7B).

The Pln J/K system formed a significantly larger pore than the Pln NC8-α/β system. This suggests that while both are effective, Pln J/K might induce more rapid cellular depolarization due to the larger diameter of the water-conducting channel. The peptides were grouped as two-peptide systems because they constitute Class IIb bacteriocins. The simulations confirmed that these pairs co-localize and cooperate to form functional pores, supporting their classification. The use of a 2:1 POPE:POPG ratio provided a representative bacterial environment. The pore formation observed in the simulation suggests a possible membrane-disruption mechanism for the predicted plantaricin peptides. However, these results are based on computational modeling and do not confirm the production or antimicrobial activity of these peptides in the studied strains.

## 3. Discussion

MDR *P. aeruginosa* remains a major global clinical concern and is implicated in severe infections, including pneumonia, bloodstream infections, UTI, and wound infections [3]. The high prevalence of antimicrobial resistance in this pathogen has substantially diminished the efficacy of first-line antibiotics, such as carbapenems, fluoroquinolones, and aminoglycosides, resulting in high rates of treatment failure and recurrent infection [5]. These concerns raise the urgent need for alternative treatments, such as probiotics, bacteriocins, antimicrobial peptides, phage therapy, and postbiotic compounds, to support or replace traditional therapies [20]. In this study, we successfully isolated five *L. plantarum* strains from fermented cacao beans that effectively inhibit MDR *P. aeruginosa*.

A specific microbial habitat with significant biological potential is represented by fermented cocoa. The mucilaginous pulp of *Theobroma cacao* is high in fermentable carbohydrates and organic nutrients, allowing for spontaneous fermentation once the pods are opened [13]. This process follows a characteristic microbial succession: yeasts initiate sugar metabolism, producing ethanol and early flavor substances, LAB subsequently dominate and convert substrates into lactic acid, and acetic acid bacteria subsequently oxidize ethanol into acetic acid [21]. The stress-laden environment created by these metabolite shifts, including high acidity and ethanol accumulation, likely serves as a strong selective pressure, favoring LAB strains with robust stress-tolerance mechanisms. LAB commonly detected during cacao bean fermentation include *Fructobacillus pseudoficulneus*, *Fructobacillus tropaeoli*, *Leuconostoc pseudomesenteroides*, and *L. plantarum* [21].

In this study, five LAB isolates obtained from fermented cacao beans in southern Thailand were identified as *L. plantarum*, which has a long history of safe use in food fermentation, contributing to improved sensory quality, preservation, and nutritional value. It is classified as GRAS and is included on the European Food Safety Authority’s (EFSA) Qualified Presumption of Safety (QPS) list, underscoring its strong safety profile [22]. Previous research has demonstrated that *L. plantarum* RUB1, isolated from koumiss-fermented yogurt, produces a class IIb bacteriocin exhibiting strong antibacterial activity, effectively inhibiting *E. coli*, *B. subtilis*, *B. cereus*, and *S. aureus* [23]. Another study reported that *L. plantarum* B21, isolated from Vietnamese fermented sausage (*nem chua*), exhibits broad-spectrum antimicrobial activity, particularly against Gram-positive foodborne pathogens [24]. Consistent with previous reports, all isolates in this study exhibited strong inhibitory activity against MDR *P. aeruginosa*. Genome analysis identified multiple bacteriocin gene clusters, including plantaricin A, J, K, N, F, E, and the NC8 variants commonly associated with antimicrobial activity in *L. plantarum*. Bacteriocins produced by this species are known to disrupt bacterial membrane integrity and interfere with quorum-sensing pathways, thereby reducing virulence and biofilm formation [16]. Plantaricins are divided into two major classes: Class I (post-translationally modified) and Class II (unmodified) peptides, both of which possess amphiphilic structures that enable binding to the negatively charged surfaces of Gram-negative bacteria. After electrostatic attachment, their hydrophobic domains insert into the lipid bilayer and oligomerize to form pores, resulting in membrane permeabilization, leakage of intracellular contents, and eventual cell death [16,25].

Our molecular dynamics simulations suggest that plantaricin peptides may interact with bacterial phospholipid bilayers and potentially form pore-like structures once associated with the membrane. However, Gram-negative bacteria such as *P. aeruginosa* possess an outer membrane containing lipopolysaccharides, which may limit the access of bacteriocins to the inner membrane. Therefore, the simulations illustrate a possible membrane-disruption mechanism rather than confirming direct activity against Gram-negative membranes. Furthermore, although bacteriocin-related genes were identified in the genomes of the studied strains, their presence does not necessarily confirm gene expression or direct involvement in the observed antimicrobial activity. The variation in inhibition zone diameters among the five *L. plantarum* isolates may reflect differences in metabolite production or regulatory mechanisms controlling gene expression. The inhibitory effects observed in the agar well diffusion assay are therefore likely associated with multiple antimicrobial metabolites produced by *L. plantarum*, including organic acids, hydrogen peroxide, and potentially bacteriocin-related peptides. Further studies are required to clarify the relationship between genomic potential and phenotypic antimicrobial activity. In addition to their antimicrobial activity, the five *L. plantarum* isolates exhibited key probiotic traits. All strains survived acidic pH, pepsin, pancreatin, and bile salts, consistent with the presence of stress-response genes such as *dnaK*, *dnaJ*, *clpB*, *cspLA*, and *tpx*, which contribute to tolerance under gastrointestinal and environmental stresses. Strong adhesion to Caco-2 cells, supported by SEM observations, corresponded with adhesion-related genes including *dltA*/*dltD*, which influence cell surface charge and promote host interaction [26]. Notably, four strains carried the quorum-sensing gene *luxS*, associated with biofilm formation and epithelial colonization, whereas CR02 lacked this gene, which may explain its comparatively lower adhesion capacity [26].

Moreover, safety evaluations based on WGS revealed no acquired antibiotic resistance or virulence-associated genes, and the absence of pathogenicity determinants. These findings are consistent with phenotypic data showing non-hemolytic behavior and susceptibility to clinically important antibiotics, such as erythromycin, tetracycline, and chloramphenicol [27]. The lack of plasmids and toxin-related prophages further supports the genomic stability and safety of these strains for probiotic or food-related applications. Thus, the phenotypic and genomic data confirm the probiotic potential of these cacao-derived isolates.

## 4. Materials and Methods

### 4.1. Bacterial Strains and Growth Conditions

*P. aeruginosa* ATCC 15692 and 17 clinical isolates of *P. aeruginosa* were used in this study. The clinical isolates were obtained from Songklanagarind Hospital, where they had previously been identified using standard microbiological methods. All strains were cultured in Brain Heart Infusion (BHI) broth (HiMedia, Mumbai, India) and incubated at 37 °C for 18 h. Cultures were supplemented with 25% (*v*/*v*) glycerol and stored at −80 °C until further use.

### 4.2. Collection of Fermented Cacao Beans and Isolation of *Lactiplantibacillus plantarum*

Fermented cacao bean samples were collected from local farms in Nakhon Si Thammarat Province, southern Thailand during June 2025. The samples were transported to the laboratory under sterile conditions for microbiological analysis. Lactic acid bacteria were isolated using de Man, Rogosa, and Sharpe (MRS) agar (HiMedia, Mumbai, India) and incubated at 37 °C for 24 h. Cultures were streaked onto MRS agar and incubated under the same conditions for an additional 24 h. The isolates were maintained in MRS broth (HiMedia, Mumbai, India) supplemented with 30% (*v*/*v*) glycerol and stored at −80 °C for further analyses.

### 4.3. Phenotypic and MALDI-TOF Identification

Gram staining, microscopic observation, and catalase activity tests were performed according to the procedures described in Bergey’s Manual of Systematic Bacteriology [28]. The bacterial identities were further confirmed using a matrix-assisted laser desorption/ionization time-of-flight mass spectrometry (MALDI-TOF MS) BioTyper system (Bruker Daltonics, Karlsruhe, Germany) following the manufacturer’s instructions. Briefly, 3–5 fresh colonies were suspended in 300 µL of sterile water and mixed with 900 µL of absolute ethanol. The mixture was centrifuged, and the pellet was resuspended in formic acid and incubated for 30 min. Then, an equal volume of 100% acetonitrile was added, and the supernatant was collected. For analysis, 2 µL of the supernatant was mixed with 1 µL of α-cyano-4-hydroxycinnamic acid (HCCA) matrix solution (10 mg/mL in a solvent composed of 5% trifluoroacetic acid and 100% acetonitrile, 1:1 *v*/*v*) and spotted onto a MALDI target plate. Spectra were acquired using a Bruker Ultraflex III TOF/TOF mass spectrometer operated in positive linear mode over a mass range of 2–20 kDa. Data were processed and analyzed with the BioTyper 2.0 software using standard quality control parameters.

### 4.4. Inhibition of P. aeruginosa by L. plantarum Strains

The antimicrobial activity of *five L. plantarum* strains (CR02, CR09, CR10, CR13, and CR14) against *P. aeruginosa* was evaluated using the agar well diffusion method according to the method reported by Nigam et al. [29], with slight modifications. Briefly, *P. aeruginosa* ATCC 15692 and 17 clinical isolates were cultured in BHI broth, while *L. plantarum* strains were cultured in MRS broth. All cultures were incubated at 37 °C for 18 h. The bacterial suspensions of *P. aeruginosa* were adjusted to a turbidity equivalent to the 0.5 McFarland standard and spread onto Mueller-Hinton agar (MHA) plates (HiMedia, Mumbai, India). Wells of 6 mm diameter were aseptically punched into the agar using the back of a sterile pipette tip. *Five L. plantarum* strains were centrifuged at 8000× *g* for 10 min, and the supernatants were collected and filtered through a 0.22 µm membrane filter to obtain sterile cell-free supernatants (CFS). Subsequently, 100 µL of the CFS was added to each well to evaluate antimicrobial activity against *Pseudomonas aeruginosa*. MRS broth served as a negative control. Plates were incubated at 37 °C for 24 h. Results were interpreted based on the criterion that a zone of inhibition greater than the 6 mm well diameter indicates antimicrobial activity. All experiments were performed in triplicate, and the results were expressed as mean ± standard deviation (SD).

### 4.5. Characterization of Probiotic Properties

#### 4.5.1. Tolerance to Low pH

*L. plantarum* strains were cultured in MRS broth and incubated at 37 °C for 24 h. One mL of each culture was harvested and centrifuged at 5000× *g* for 5 min. The resulting pellets were washed twice with phosphate-buffered saline (PBS; Sigma-Aldrich, St. Louis, MO, USA) and adjusted to a turbidity equivalent to the 0.5 McFarland standard. The cell suspensions were then centrifuged again at 5000× *g* for 5 min to collect the pellets, which were resuspended in MRS broth adjusted to pH 2, 3, 4, and 7. The mixtures were incubated at 37 °C for 3 h to evaluate acid tolerance. Viable cell counts were determined by spreading serial dilutions on MRS agar plates, and the survival rate (%) was calculated using the following equation:

$$Survival\;rate\;(\%)=\frac{Final(LogCFU/mL)}{Initial(LogCFU/mL)}\times 100$$

#### 4.5.2. Tolerance to Pepsin, Pancreatin, and Bile Salt

The tolerance of *L. plantarum* strains to gastrointestinal conditions was evaluated by assessing their resistance to pepsin, pancreatin, and bile salts. Tolerance to pepsin and pancreatin digestion was evaluated following the method of Monteagudo-Mera et al. [30], with slight modifications. Pepsin solution was prepared by dissolving pepsin (Sigma-Aldrich, St. Louis, MO, USA) in MRS broth at a concentration of 3 g/L, and the pH was adjusted to 2.0 with HCl. Pancreatin solution was prepared by suspending pancreatin (Sigma-Aldrich, St. Louis, MO, USA) in MRS broth at a concentration of 1 g/L and adjusting the pH to 8.0 with 0.1 M NaOH. One mL of each overnight culture was centrifuged at 5000× *g* for 5 min, washed with PBS, and resuspended in MRS broth containing either pepsin or pancreatin. The suspensions were incubated at 37 °C for 3 h (pepsin) or 4 h (pancreatin). Viable colonies were enumerated on MRS agar before and after enzyme treatment, and the survival rate (%) was calculated as described above.

Bile salt tolerance was assessed according to the method of Monteagudo-Mera et al. [30], with modifications. Overnight cultures of *L. plantarum* were harvested by centrifugation at 5000× *g* for 5 min and resuspended in MRS broth containing 0.3% (*w*/*v*) bile salts (Sigma-Aldrich, St. Louis, MO, USA). The cultures were incubated at 37 °C for 4 h, and viable counts were determined on MRS agar plates. The percentage of surviving cells was calculated using the same formula.

#### 4.5.3. Auto-Aggregation Assay

Auto-aggregation ability of *L. plantarum* strains was evaluated according to the method of Somashekaraiah et al. [31], with minor modifications. Briefly, overnight cultures were harvested by centrifugation at 5000× *g* for 5 min and washed twice with PBS (pH 7.2). The pellets were resuspended in the same buffer and incubated anaerobically at 37 °C. The optical density (OD) of the upper suspension was measured at 600 nm at different time intervals (0, 2, 4, and 24 h). The percentage of auto-aggregation was calculated using the following equation:

$$Auto\;aggregation\;\%=[1-(\frac{A_{time}}{A_{0}})]\times 100$$

where A_time_ is the absorbance at a particular time, and A_0_ is the absorbance at time 0.

#### 4.5.4. Cell Surface Hydrophobicity Assay

Cell surface hydrophobicity was assessed using the xylene partitioning method described by Vlkova et al. [32]. Bacterial cells were harvested, washed, and resuspended in PBS (pH 7.2) to an initial absorbance (A_0_) of approximately 0.5 at 600 nm. Three milliliters of bacterial suspension was mixed with 1 mL of xylene, vortexed for 2 min, and allowed to stand for phase separation at room temperature for 30 min. The aqueous phase was carefully collected, and its absorbance (A) was measured at 600 nm. The hydrophobicity (%) was calculated as follows:

$$H\%=(\frac{A_{0}-A}{A_{0}})\times 100$$

where A_0_ and A are absorbance values measured before and after xylene extraction, respectively.

#### 4.5.5. Adhesion to the Caco-2 Cell Line

The adhesion ability of *L. plantarum* strains was evaluated using human colorectal adenocarcinoma (Caco-2) cells purchased from ATCC. The protocol was adapted from Zawistowska-Rojek et al. [33] with minor modifications. Caco-2 cells were cultured in Dulbecco’s Modified Eagle Medium (DMEM; Thermo Fisher Scientific, Waltham, MA, USA) supplemented with 3 mM L-glutamine and antibiotics (50 µg/mL penicillin-streptomycin, 50 µg/mL gentamicin, and 1.25 µg/mL amphotericin B). The cells were seeded into 24-well tissue culture plates and incubated at 37 °C in a humidified atmosphere containing 5% CO_2_ until reaching 85–90% confluence to form a monolayer. Bacterial suspensions were prepared at a concentration of approximately 10^8^ CFU/mL and added to each well. After incubation for 2 h at 37 °C under 5% CO_2_, non-adherent bacteria were removed by washing the monolayers four times with sterile PBS. Then, 200 µL of 2.5% (*w*/*v*) trypsin solution was added to disaggregate the cell monolayer, followed by 800 µL of complete DMEM. The resulting suspension containing the released bacteria was serially diluted and plated on MRS agar for enumeration.

The adhesion ability (%) was calculated using the following equation:

$$\%\;Adhesion\;ability=\frac{V_{1}\times 100}{V_{0}}$$

where V_0_ is the initial viable count and V_1_ is the viable count adhered to the Caco-2 cells after incubation

#### 4.5.6. SEM of *L. plantarum* Adhered to Caco-2 Cells

The adhesion of *L. plantarum* strains to Caco-2 cells was visualized by SEM following the procedure of Talib et al. [34], with slight modifications. After the adhesion assay, Caco-2 cell monolayers were fixed with 2.5% (*v*/*v*) glutaraldehyde (Sigma-Aldrich, St. Louis, MO, USA) prepared in 0.1 M phosphate buffer (pH 7.2) for 24 h at 4 °C. The fixed samples were then dehydrated through a graded ethanol series (40%, 60%, 80%, and 95% *v*/*v*), each for 15 min, followed by two dehydration steps in 100% ethanol (Fisher Scientific, Fairlawn, NJ, USA) for 15 min each.

Coverslips containing the dehydrated cells were air-dried at room temperature for 30 min, mounted on aluminum stubs, and sputter-coated with a thin layer of gold for 3 min. The surface morphology and bacterial adhesion on Caco-2 cells were examined using a field-emission scanning electron microscope (Merlin Compact, Zeiss, Oberkochen, Germany) equipped with energy-dispersive X-ray spectroscopy (EDX; Aztec, Oxford, UK) and electron backscatter diffraction (EBSD; Nordlys Max, Oxford, UK)

#### 4.5.7. Hemolytic Activity

The hemolytic potential of *L. plantarum* isolates was evaluated using blood agar (BA) plates (HiMedia, Mumbai, India) following the method described by Leite et al. [35]. Overnight cultures of each isolate were streaked onto BA plates and incubated at 37 °C for 24 h. Hemolytic activity was evaluated by the presence and type of clear zones surrounding bacterial colonies, indicating β-hemolysis (complete lysis), α-hemolysis (partial lysis), or γ-hemolysis (no lysis).

#### 4.5.8. Antibiotic Susceptibility

Antibiotic susceptibility testing was performed according to the guidelines of the Clinical and Laboratory Standards Institute (CLSI 2025) [36]. The disk diffusion method was used to evaluate resistance profiles against a panel of antibiotics (Oxoid, Hampshire, UK), including ampicillin (10 µg), vancomycin (30 µg), gentamicin (10 µg), erythromycin (15 µg), clindamycin (2 µg), tetracycline (30 µg), kanamycin (30 µg), chloramphenicol (30 µg), and streptomycin (10 µg). Plates were incubated at 37 °C for 24 h, and the diameters of inhibition zones were measured in millimeters. The isolates were classified as susceptible, intermediate, or resistant according to CLSI interpretive criteria.

#### 4.5.9. Bacteriocin Activity Screening and pH-Neutralized Assay

To distinguish between acid-mediated and proteinaceous antimicrobial effects, cell-free supernatants (CFS) from overnight cultures of *L. plantarum* were obtained by centrifugation (7000× *g*, 10 min). Three conditions were evaluated: (i) untreated CFS, (ii) pH-neutralized CFS (pH 7.0, 1 N NaOH), and (iii) CFS treated with proteinase K (1 mg/mL, 30 °C, 120 min) followed by heat inactivation (80 °C, 10 min) [37]. All samples were filter-sterilized (0.2 μm). Antimicrobial activity against *P. aeruginosa* was determined using the agar well diffusion method as described in Section 4.4.

### 4.6. DNA Extraction and Genomic Analysis

Genomic DNA from the five *L. plantarum* strains (CR02, CR09, CR10, CR13, and CR14) was extracted using the DNeasy Blood & Tissue Kit (QIAGEN, Hilden, Germany), following the manufacturer’s protocol. The concentration and purity of the extracted DNA were assessed spectrophotometrically by calculating the A260/A280 ratio and confirmed by agarose gel electrophoresis.

Genomic DNA was submitted to the Beijing Genomics Institute (BGI, China) for WGS using the MGISEQ-2000 platform (MGI Tech Co., Ltd., Shenzhen, China) with 150 bp paired-end reads. The raw sequencing data were processed using the BacSeq v1.0 pipeline [38], an integrated workflow that performs assembly, annotation, and quality assessment through established bioinformatics tools, including SPAdes v4.1.0 for genome assembly [39], Prokka v1.2.0 for gene annotation [40], QUAST for assembly quality metrics [41], and BUSCO v6.0.0 for genome completeness evaluation [42]. Mobile genetic elements, prophage regions, and ARGs were identified using mobileOG-db v1.1.3 [43], PHASTEST v2.0.14 [44], VirulenceFinder v2.0.5 [45], and ResFinder v4.7.2 [46], respectively. ARG detection was based on a ≥90% sequence identity threshold and a ≥60% minimum alignment length. CRISPR-Cas systems were characterized using CRISPRCasFinder v4.2.20 [47]. In addition, genes encoding ribosomally synthesized and post-translationally modified peptides, including bacteriocins, were predicted using the BAGEL4 web server [48]. The Roary pipeline [49] was used for pan-genome analysis with a 95% BLASTp v2.16.0 cutoff to group genes into core, accessory, and unique clusters. Comparative genomics was performed with Proksee 1.0.0a6 [50] and BLASTn v2.16.0 [51] to assess coding sequence similarity, and genomic relatedness was further evaluated using OrthoANI v0.7.0 [52] for average nucleotide identity analysis.

### 4.7. Computational Methods

#### 4.7.1. Peptide Preparation

The peptide structures of plantaricin J, plantaricin K, plantaricin NC8-α, and plantaricin NC8-β (see details in Supplementary Table S1) were predicted using the AlphaFold 3.0 web server (https://alphafoldserver.com) [53]. The peptide sequences were obtained by translating the bacteriocin genes identified in the genomes of the studied *Lactiplantibacillus plantarum* strains, and only the core peptide sequences corresponding to the mature bacteriocins were used for structural prediction. According to previous studies [54,55], these bacteriocins were selected based on their documented antimicrobial activity and genetic characterization in *L. plantarum*. Plantaricin J and plantaricin K (Pln J/K), as well as plantaricin NC8-α and plantaricin NC8-β (Pln NC8-α/β), were analyzed as two-peptide (class IIb) bacteriocin systems.

#### 4.7.2. System Preparation

CG molecular dynamics systems were generated using CHARMM-GUI [54] with the Martini 3.0.0 force field [56]. All peptide termini were modeled in their charged states, with protonated N-termini and deprotonated C-termini. Two peptide systems were prepared corresponding to the two-peptide bacteriocin pairs: Plantaricin J and Plantaricin K (Pln J/K), and Plantaricin NC8-α and Plantaricin NC8-β (Pln NC8-α/β). For each system, a total of 10 peptides were included, consisting of equal numbers of each peptide within the pair (i.e., five molecules of each peptide). The peptides were initially placed in a transmembrane orientation, representing a post-insertion state in which the peptides had passed through and were embedded within the membrane. Individual peptides were positioned such that the center-of-mass distance between neighboring peptides was approximately 30 Å, minimizing initial steric interactions. Each system consisted of a lipid bilayer, peptides, water, and counterions. The lipid bilayer was composed of POPE and POPG at a 2:1 ratio, consistent with bacterial membrane models reported previously [57,58]. The membrane dimensions along the x- and y-axes were approximately 220 Å, and the bilayer was solvated with 22.5 Å of water on both the upper and lower leaflets. The system was neutralized and ionized with Na^+^ and Cl^−^ ions to achieve a physiological salt concentration of 0.15 M NaCl.

#### 4.7.3. CG Simulation and Visualization

All CG molecular dynamics simulations were performed using GROMACS 2024.5 [54]. To achieve a total sampling time of 4 μs, the production simulations were executed in sequential segments, enabling efficient checkpointing, restart capability, and data management while maintaining trajectory continuity. Computational performance was optimized using a hybrid CPU/GPU acceleration scheme. Bonded and non-bonded interactions were offloaded to the GPU, while long-range electrostatics were computed on CPU cores using OpenMP threading with either the Particle Mesh Ewald (PME) or Reaction-Field approach, as appropriate for the CG model.

Prior to production runs, all systems underwent energy minimization to eliminate steric clashes and unfavorable contacts, followed by multi-stage equilibration to relax the system. During equilibration, temperature was maintained using the v-rescale thermostat, and pressure was controlled using the Berendsen barostat. Position restraints were applied to peptide backbones and lipid headgroups and were gradually reduced from 1000 to 50 kJ mol^−1^ nm^−2^ to allow a smooth relaxation of the system while preserving structural integrity. Production simulations were carried out for a total duration of 4 μs using the Parrinello–Rahman barostat with semi-isotropic pressure coupling at 1.0 bar, appropriate for membrane systems. The system temperature was maintained at 310 K using the v-rescale thermostat. A 20 fs integration time step was employed, consistent with the stability limits of the Martini 3 force field. The production phase was executed as multiple consecutive runs, with each segment initialized from the final coordinates and velocities of the preceding segment to ensure uninterrupted sampling.

To address the challenges of visualizing CG resolution systems in a software designed for atomic-level detail, the MartiniGlass Python package v1.1.3 [59] was employed. MartiniGlass facilitates the visualization of systems simulated with the Martini force field by rapidly processing molecular topologies and accounting for critical structural features, such as secondary structure restraints. In this study, the tool was used to map implicit CG interactions into a visualizable format compatible with VMD (Visual Molecular Dynamics) [60], ensuring molecular integrity across periodic boundaries. Specifically, the protein backbone (BB) beads were manually assigned a helical configuration using VMD’s internal structure commands to accurately reflect the secondary structure restraints processed by MartiniGlass.

### 4.8. Statistical Analysis

All data are presented as mean ± SD from at least three independent experiments. Statistical significance between groups was assessed using one-way analysis of variance, followed by post hoc testing where appropriate, or by Student’s *t*-test for pairwise comparisons. A *p*-value < 0.05 was considered statistically significant.

## 5. Conclusions

To the best of our knowledge, this study is the first to identify and characterize *L. plantarum* strains from fermented cacao beans in Thailand with strong anti-*P. aeruginosa* activity and promising probiotic properties. Our findings bridge the gap between food microbiology and clinical therapeutics, highlighting that fermented cacao beans serve as a valuable reservoir of novel probiotic candidates with bacteriocin-mediated antimicrobial potential. Future studies should focus on isolating and purifying specific plantaricins, determining their antimicrobial activity spectra, and experimentally confirming their biological activity. In addition, in vivo studies are needed to evaluate their potential applications in functional foods or as adjunct strategies for managing MDR pathogens.

## Figures and Tables

**Table 1:** Antimicrobial activity of *Lactiplantibacillus plantarum* isolated from fermented cacao beans (Inhibition zone diameter, mm).

Bacteria	CR02	CR09	CR10	CR13	CR14
*P. aeruginosa* ATCC 15692	17.67 ± 0.58	20.67 ± 0.58	20.00 ± 1.73	18.00 ± 0.00	21.00 ± 0.00
*P. aeruginosa* 532610	16.67 ± 0.58	20.33 ± 0.58	20.00 ± 0.00	20.00 ± 0.00	19.67 ± 0.58
*P. aeruginosa* 4293-04	20.33 ± 0.58	18.00 ± 0.00	18.00 ± 0.00	19.33 ± 0.58	19.00 ± 0.00
*P. aeruginosa* 3285-10	18.67 ± 0.58	22.33 ± 1.15	20.67 ± 0.58	20.33 ± 0.58	20.67 ± 0.58
*P. aeruginosa* 479-11	16.33 ± 0.58	20.33 ± 0.58	16.67 ± 0.58	18.33 ± 0.58	19.33 ± 0.58
*P. aeruginosa* 2910-10	20.33 ± 0.58	22.00 ± 0.00	20.33 ± 0.58	20.33 ± 0.58	20.00 ± 0.00
*P. aeruginosa* 1032-10	16.67 ± 0.58	17.67 ± 0.58	18.00 ± 1.00	18.33 ± 0.58	18.33 ± 0.58
*P. aeruginosa* 1533-04	21.67 ± 0.58	19.67 ± 0.58	18.33 ± 0.58	19.33 ± 0.58	17.33 ± 0.58
*P. aeruginosa* 4591-10	18.33 ± 0.58	20.33 ± 0.58	21.33 ± 0.58	18.67 ± 0.58	18.33 ± 0.58
*P. aeruginosa* 2285-10	20.33 ± 0.58	20.33 ± 0.58	18.33 ± 0.58	20.00 ± 0.00	20.33 ± 0.58
*P. aeruginosa* 3685-10	18.67 ± 0.58	21.00 ± 0.00	21.67 ± 058	19.7 ± 0.58	20.67 ± 0.58
*P. aeruginosa* 730-10	20.00 ± 0.00	18.33 ± 0.58	18.33 ± 0.58	20.00 ± 0.00	19.33 ± 0.58
*P. aeruginosa* 4135-03	19.33 ± 0.58	20.33 ± 0.58	21.00 ± 0.00	20.00 ± 1.00	20.00 ± 0.00
*P. aeruginosa* 4712-04	17.67 ± 0.58	18.33 ± 0.58	16.33 ± 0.58	18.67 ± 0.58	20.67 ± 0.58
*P. aeruginosa* 4260-03	17.67 ± 0.58	17.33 ± 0.58	18.33 ± 0.58	20.33 ± 058	20.67 ± 0.58
*P. aeruginosa* 1918-10	22.00 ± 0.00	20.33 ± 0.58	21.33 ± 0.58	24.00 ± 0.00	21.33 ± 0.58
*P. aeruginosa* 1091-10	20.33 ± 0.58	20.33 ± 0.58	18.33 ± 0.58	19.67 ± 0.58	20.33 ± 0.58
*P. aeruginosa* 1450-11	19.33 ± 0.58	18.33 ± 0.58	19.33 ± 0.58	19.00 ± 0.00	19.33 ± 0.58

Data are expressed as mean ± standard deviation (SD) of three independent experiments.

**Table 2:** Survival rate (%) of *Lactiplantibacillus plantarum* in GIT conditions.

Isolates	pH 2	pH 3	pH 4	pH 7	Pepsin	Pancreatin	0.3% Bile Salts
CR02	29.67 ±3.04	29.57 ± 5.07	80.65 ± 5.38	90.08 ± 8.73	96.12 ± 1.20	91.37 ± 0.72	98.92 ± 0.75
CR09	12.78 ±4.10	17.36 ± 5.98	21.12 ± 1.11	98.61 ± 2.40	93.84 ± 0.16	99.39 ± 0.26	98.98 ± 0.80
CR10	17.22 ± 2.55	19.09 ± 2.05	55.72 ± 8.90	96.22 ± 0.42	92.50 ± 0.07	99.88 ± 0.21	92.79 ± 1.03
CR13	14.38 ±5.00	15.89 ± 7.03	71.18 ± 7.64	91.53 ± 7.50	88.08 ± 1.46	99.75 ± 0.07	98.51 ± 1.29
CR14	61.15 ± 7.75	27.32 ± 9.94	41.63 ± 5.66	83.33 ± 5.56	89.65 ± 1.08	99.78 ± 0.21	98.35 ± 1.00

Data are expressed as mean ± standard deviation (SD) of three independent experiments.

**Table 3:** Main genome features of *Lacticaseibacillus plantarum* strains.

Feature	CR02	CR09	CR09	CR13	CR13
Total length	3,465,846	3,233,809	3,232,214	3,291,372	3,291,974
GC (%)	44.14	44.48	44.48	44.41	44.41
N50	101,367	421,222	421,222	195,932	483,721
L50	12	3	4	4	3
Number of contigs	154	21	26	36	30
CDS	3311	3039	3036	3119	3121
rRNA	2	4	4	4	3
tRNA	50	60	58	61	61
tmRNA	1	1	1	1	1
Prophage	8	5	5	5	5
Bacteriocin-like encoding gene	6	4	4	6	6

**Table 4:** Genes associated with probiotic functions of five *Lacticaseibacillus plantarum* strains.

Function	Gene	CR02	CR09	CR10	CR13	CR14
**Gastrointestinal tract survival**	*pbpB*	+	+	+	+	+
	*penA*	+	+	+	+	+
	*pbpE*	-	-	-	-	-
	*ponA*	+	+	+	+	+
	*pbpF_1*	-	-	-	-	-
	*pbpF_2*	-	-	-	-	-
	*pbpX*	+	-	-	-	-
	*pbp*	-	-	-	-	-
**Acid tolerance**	*nhaK_2*	+	+	+	+	+
	*atpA*	+	+	+	+	+
	*atpF*	+	+	+	+	+
	*atpG*	+	+	+	+	+
	*atpB*	+	+	+	+	+
	*atpD*	+	+	+	+	+
	*atpH*	+	+	+	+	+
	*atpE*	+	+	+	+	+
**Bile salt tolerance**	*murE*	+	+	+	+	+
	*mleS*	+	+	+	+	+
**Temperature tolerance**	*cspB*	-	-	-	-	-
	*cspLA*	+	+	+	+	+
	*csp*	+	+	+	+	+
	*hrcA*	+	+	+	+	+
	*dnaJ*	+	+	+	+	+
	*dnaK*	+	+	+	+	+
	*clpC_1*	+	+	+	+	+
	*clpB*	+	+	+	+	+
**Osmotic shock tolerance**	*grpE*	+	+	+	+	+
	*gbuA*	-	-	-	-	-
	*gbuC*	-	-	-	-	-
	*gbuB*	-	-	-	-	-
	*opuCD*	+	+	+	+	+
	*opuCC*	+	+	+	+	+
**Oxidative stress survival**	*hslO*	+	+	+	+	+
	*nox_2*	+	+	+	+	+
	*nox_1*	+	+	+	+	+
	*tpx*	+	+	+	+	+
	*npr*	-	-	-	-	-
**Cell wall formation**	*murA1*	+	+	+	+	+
	*epsH_2*	-	-	-	-	-
	*ykoT_1*	-	-	-	-	-
	*tagE*	+	+	+	-	-
	*dltC*	-	-	-	-	-
	*dltA*	+	+	+	+	+
	*dltD*	+	+	+	+	+
	*dltC*	-	-	-	-	-
**Biofilm formation**	*ywqC*	-	-	-	-	-
	*luxS*	-	+	+	+	+
	*desR*	+	+	+	+	+
	*ccpA_2*	+	+	+	+	+
	*brpA_2*	-	-	-	-	-
	*brpA_4*	-	-	-	-	-
	*brpA_3*	-	-	-	-	-
**Vitamin synthesis**	*btuD_14*	-	-	-	-	-
	*btuD_14*	-	-	-	-	-
	*btuD_2*	+	+	+	+	+
	*btuD_8*	-	-	-	-	-
	*btuD_13*	-	-	-	-	-
	*btuD_4*	+	+	+	+	+
	*btuD_15*	-	-	-	-	-
	*btuD_5*	+	+	+	+	+
	*btuD_9*	-	-	-	-	-
	*btuD_12*	-	-	-	-	-
	*btuD_11*	-	-	-	-	-
	*btuD_7*	-	-	-	-	-
	*btuD_6*	+	+	+	+	+
	*btuD_1*	+	+	+	+	+
	*btuD_3*	+	+	+	+	+
**Bacteriocin-encoding gene**	*plnA*	+	+	-	+	+
	*plnK*	+	+	+	+	+
	*plnJ*	+	+	+	+	+
	*plnN*	+	+	-	+	+
	*plnE*	+	-	-	+	+
	*plnF*	+	-	-	+	+
	*plnNC8-β*	-	-	+	-	-
	*plnNC8-α*	-	-	+	-	-

## Data Availability

All data are included in article/Supplementary Material/references in article.

## Supplementary Materials

The following supporting information can be downloaded at: https://www.mdpi.com/article/10.3390/antibiotics15040334/s1, Table S1. Characteristics of selected plantaricin peptides. Table S2. Bruker MALDI-TOF MS Biotyper identification results of Lactiplantibacillus plantarum. Table S3. Antibiotic susceptibility of 5 Lactiplantibacillus plantarum. Table S4. Predicted prophage regions in the genome of Lacticaseibacillus paracasei strains. Figure S1. Gene cluster organization for bacteriocin biosynthesis in five Lacticaseibacillus *L. plantarum isolates*.
