# Supplementary material for: Probiotic Potential, Genomic Characterization, and In Silico Insights of Five Lactiplantibacillus plantarum Strains Isolated from Fermented Cacao Beans Against Multidrug-Resistant Pseudomonas aeruginosa
Source: Antibiotics (Basel). 2026 Mar 26;15(4):334. doi: 10.3390/antibiotics15040334 (PMC13114214; doi:10.3390/antibiotics15040334)
Supplement: Supplementary file 1 [file antibiotics-15-00334-s001.zip › antibiotics-4180263-supplementary.pdf]

**Supplementary Table S1.** Characteristics of selected plantaricin peptides

| **Bacteriocin** | Amino acid sequence (N→C) | **Length (residues)** | ***Predicted molecular weight (kDa)** |
|---|---|---|---|
| Plantaricin J | TVNKMIKDLDVVDAFAPISNNKLNGVVGGA WKNFWSSLRKGFYDGEAGRAIRR | 54 | 5.94 |
| Plantaricin K | KIKLTVLNEFEELTADAEKNISGGRRSRKNGIGY AIGYAFGAVERAVLGGSRDYNK | 56 | 6.11 |
| Plantaricin NC8-α | DKFEKISTSNLEKISGGDLTTKLWSSWGYYLGK KARWNLKHPYVQF | 46 | 5.42 |
| Plantaricin NC8-β | NNLNKFSTLGKSSLSQIEGGSVPTSVYTLGIKIL WSAYKHRKTIEKSFNKGFYH | 54 | 6.08 |

*Amino acid length and molecular weight were predicted using the GeneCorner Protein Molecular Weight Calculator (https://www.genecorner.ugent.be/protein_mw.html).

**Supplementary Table S2.** Bruker MALDI-TOF MS Biotyper identification results of *Lactiplantibacillus plantarum.*

| Isolates | Matched Pattern | Score Value |
|---|---|---|
| CR02 | *Lactobacillus plantarum* DSM 2601 DSM | 2.27 |
| CR09 | *Lactobacillus plantarum* DSM 1055 DSM | 2.21 |
| CR10 | *Lactobacillus plantarum* DSM 2601 DSM | 2.21 |
| CR13 | *Lactobacillus plantarum* DSM 13273 DSM | 2.37 |
| CR14 | *Lactobacillus plantarum* DSM 1055 DSM | 2.33 |

Meaning of Score Values: 2.00 - 3.00; High-confidence identification, 1.70 - 1.99; Low-confidence identification, 0.00 - 1.69; No organism identification possible

**Supplementary Table S3.** Antibiotic susceptibility of 5 *Lactiplantibacillus plantarum*

| **Stains** | **Ampicillin<br>(10 ug)** | **Vancomyci<br>n (30)** | **Gentamicin<br>(10)** | **Erytomyci<br>n (15)** | **Clindamyci<br>n (2)** | **Tetracyclin<br>e (30)** | **Kanamyci<br>n (30)** | **Chloramphenico<br>l (30)** | **Streptomyci<br>n (10)** |
|---|---|---|---|---|---|---|---|---|---|
| CR02 | R | R | S | R | R | S | R | S | R |
| CR09 | R | R | S | R | R | S | R | S | R |
| CR10 | R | R | S | R | R | S | R | S | R |
| CR13 | R | R | S | R | R | S | R | S | R |
| CR14 | R | R | S | R | R | S | R | S | R |

R: resistant and, I: intermediate, S: susceptible

**Supplementary Table S4.** Predicted prophage regions in the genome of ***Lacticaseibacillus paracasei strains***

| Region | Region Length | Completeness | Score | Total Proteins | Region Position | Most Common Phage | GC % |
|---|---|---|---|---|---|---|---|
| *Lacticaseibacillus plantarum* CR02 | | | | | | | |
| CR02_00001,len=247766,cov=78.8,corr=0,origname=Contig_48_78.7897 | 16.3Kb | questionable | 80 | 23 | 58122-74429 | PHAGE_Strept_315.2_NC_004585(3) | 42.41 |
| CR02_00002,len=208379,cov=54.9,corr=0,origname=Contig_181_54.8907 | 34.5Kb | incomplete | 60 | 38 | 173804-208379 | PHAGE_Lactob_phig1e_NC_004305(4) | 41.10 |
| CR02_00010,len=113234,cov=71.2,corr=0,origname=Contig_173_71.2361 | 16.7Kb | incomplete | 40 | 16 | 96460-113232 | PHAGE_Lactob_Sha1_NC_019489(9) | 42.43 |
| CR02_00014,len=90758,cov=50.0,corr=0,origname=Contig_176_49.9758 | 30.7Kb | questionable | 90 | 34 | 60042-90756 | PHAGE_Oenoco_phiS13_NC_023560(15) | 42.33 |

| Region | Region Length | Completeness | Score | Total Proteins | Region Position | Most Common Phage | GC % |
|---|---|---|---|---|---|---|---|
| CR02_00023,len=48179,cov=55.0,corr=0,origname=Contig_19_55.016 | 30.3Kb | incomplete | 40 | 28 | 16726-47089 | PHAGE_Lactob_Sha1_NC_019489(9) | 43.11 |
| CR02_00028,len=35090,cov=81.9,corr=0,origname=Contig_115_81.949 | 34.3Kb | intact | 140 | 52 | 2-34352 | PHAGE_Lactob_Sha1_NC_019489(26) | 41.40 |
| CR02_00036,len=20923,cov=82.5,corr=0,origname=Contig_34_82.4716 | 16.9Kb | incomplete | 20 | 35 | 219-17198 | PHAGE_Lactob_Sha1_NC_019489(9) | 37.86 |
| CR02_00039,len=18709,cov=24.2,corr=0,origname=Contig_139_24.2303 | 3.9Kb | incomplete | 50 | 11 | 1-3983 | PHAGE_Lactob_phiAT3_NC_005893(2) | 37.21 |
| *Lacticaseibacillus plantarum* CR09 | | | | | | | |
| CR09_00001,len=831655,cov=46.4,corr=0,origname=Contig_3_46.3979 | 17Kb | incomplete | 20 | 28 | 138129-155162 | PHAGE_Lactob_LfeSau_NC_029068(7) | 39.29 |

| Region | Region Length | Completeness | Score | Total Proteins | Region Position | Most Common Phage | GC % |
|---|---|---|---|---|---|---|---|
| CR09_00001,len=831655,cov=46.4,corr=0,origname=Contig_3_46.3979 | 21.5Kb | incomplete | 60 | 24 | 158630-180141 | PHAGE_Lister_LP_101_NC_024387(9) | 43.51 |
| CR09_00013,len=44839,cov=55.9,corr=0,origname=Contig_21_55.8923_ | 19.5Kb | incomplete | 20 | 7 | 4782-24356 | PHAGE_Escher_ESCO5_NC_047776(4) | 38.00 |
| CR09_00014,len=38384,cov=307.9,corr=0,origname=Contig_12_307.857_ | 37.1Kb | intact | 150 | 57 | 388-37500 | PHAGE_Lactob_phig1e_NC_004305(25) | 42.62 |
| CR09_00015,len=34136,cov=55.4,corr=0,origname=Contig_13_55.3972_ | 32.5Kb | intact | 150 | 45 | 440-33001 | PHAGE_Lactob_Sha1_NC_019489(9) | 43.11 |
| *Lacticaseibacillus plantarum* CR10 | | | | | | | |
| CR10_00001,len=557942,cov=45.1,corr=0,origname=Contig_4_45.0536 | 17Kb | incomplete | 20 | 29 | 59605-76638 | PHAGE_Lactob_LfeSau_NC_029068(7) | 39.29 |

| Region | Region Length | Completeness | Score | Total Proteins | Region Position | Most Common Phage | GC % |
|---|---|---|---|---|---|---|---|
| CR10_00001,len=557942,cov=45.1,corr=0,origname=Contig_4_45.0536 | 21.5Kb | incomplete | 60 | 24 | 80106-101617 | PHAGE_Lister_LP_101_NC_024387(9) | 43.51 |
| CR10_00003,len=425592,cov=118.2,corr=0,origname=Contig_17_118.173 | 40.1Kb | intact | 150 | 58 | 385338-425441 | PHAGE_Lactob_phig1e_NC_004305(26) | 42.85 |
| CR10_00015,len=33525,cov=53.0,corr=0,origname=Contig_13_52.9911 | 33Kb | intact | 150 | 46 | 440-33525 | PHAGE_Lactob_Sha1_NC_019489(9) | 43.15 |
| CR10_00019,len=19276,cov=59.1,corr=0,origname=Contig_6_59.1096 | 7Kb | incomplete | 20 | 7 | 375-7427 | PHAGE_Escher_vB_EcoM_Schickermooser_NC_048196(4) | 38.13 |
| *Lacticaseibacillus plantarum* CR13 | | | | | | | |
| WU0801_00002,len=504667,cov=47.8,corr=0,origname=Contig_38_47.8041 | 7Kb | incomplete | 20 | 7 | 486-7538 | PHAGE_Escher_ESCO13_NC_047770(4) | 38.07 |

| Region | Region Length | Completeness | Score | Total Proteins | Region Position | Most Common Phage | GC % |
|---|---|---|---|---|---|---|---|
| WU0801_00003,len=289223,cov=45.1,corr=0,origname=Contig_22_45.1253 | 13.9Kb | questionable | 80 | 18 | 274637-288580 | PHAGE_Staphy_IME_SA4_NC_029025(2) | 41.24 |
| WU0801_00017,len=46875,cov=120.8,corr=0,origname=Contig_3_120.812 | 43.3Kb | intact | 110 | 66 | 220-43562 | PHAGE_Oenoco_phi9805_NC_023559(15) | 41.88 |
| WU0801_00019,len=40683,cov=212.4,corr=0,origname=Contig_2_212.423 | 40.5Kb | intact | 150 | 54 | 164-40682 | PHAGE_Lactob_Sha1_NC_019489(37) | 40.50 |
| WU0801_00020,len=39422,cov=1310.7,corr=0,origname=Contig_1_1310.74 | 37.4Kb | intact | 130 | 52 | 585-38083 | PHAGE_Lister_LP_101_NC_024387(8) | 41.40 |
| *Lacticaseibacillus plantarum* CR14 | | | | | | | |
| WU0802_00002,len=504667,cov=47.1,corr=0,origname=Contig_30_47.1421 | 7Kb | incomplete | 20 | 7 | 486-7538 | PHAGE_Entero_phi92_NC_023693(4) | 38.07 |

| Region | Region Length | Completeness | Score | Total Proteins | Region Position | Most Common Phage | GC % |
|---|---|---|---|---|---|---|---|
| WU0802_00003,len=483721,cov=50.5,corr=0,origname=Contig_20_50.5467 | 30.4Kb | intact | 100 | 25 | 266654-297140 | PHAGE_Strept_315.2_NC_004585(2) | 40.55 |
| WU0802_00003,len=483721,cov=50.5,corr=0,origname=Contig_20_50.5467 | 37.7Kb | intact | 100 | 49 | 295067-332807 | PHAGE_Oenoco_phi9805_NC_023559(15) | 42.53 |
| WU0802_00012,len=40683,cov=258.9,corr=0,origname=Contig_2_258.917 | 40.5Kb | intact | 150 | 54 | 164-40682 | PHAGE_Lactob_Sha1_NC_019489(37) | 40.50 |
| WU0802_00013,len=39422,cov=1332.9,corr=0,origname=Contig_1_1332.89 | 37.4Kb | intact | 130 | 52 | 585-38083 info_out line | PHAGE_Lister_B025_NC_009812(8) | 41.40 |

CR02

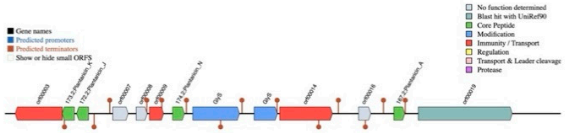


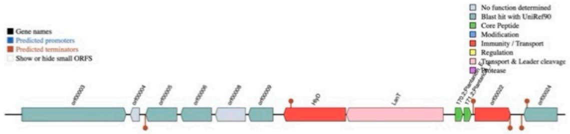


CR09

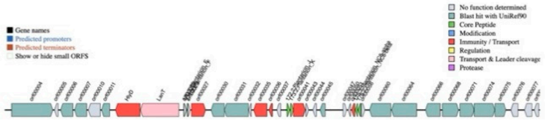


CR10

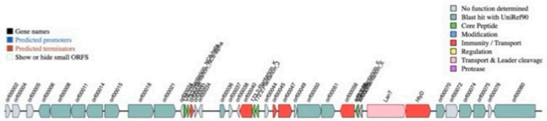


CR13

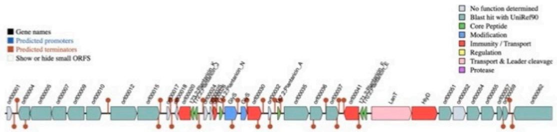


CR14

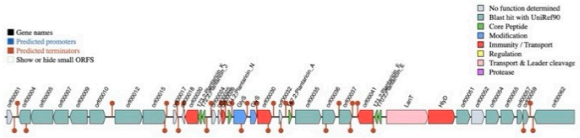

**Supplementary Figure S1.** Gene cluster organization for bacteriocin biosynthesis in five *Lacticaseibacillus L. plantarum isolates*. The cluster contains genes encoding core peptides, modification enzymes, transport, immunity, and regulatory proteins predicted using BAGEL analysis.
